# Novel Approaches for the Early Detection of Glaucoma Using Artificial Intelligence

**DOI:** 10.3390/life14111386

**Published:** 2024-10-28

**Authors:** Marco Zeppieri, Lorenzo Gardini, Carola Culiersi, Luigi Fontana, Mutali Musa, Fabiana D’Esposito, Pier Luigi Surico, Caterina Gagliano, Francesco Saverio Sorrentino

**Affiliations:** 1 Department of Ophthalmology, University Hospital of Udine, 33100 Udine, Italy; 2 Unit of Ophthalmology, Department of Surgical Sciences, Ospedale Maggiore, 40100 Bologna, Italydr.fsorrentino@gmail.com (F.S.S.); 3 Ophthalmology Unit, Department of Surgical Sciences, IRCCS Azienda Ospedaliero, Alma Mater Studiorum University of Bologna, 40100 Bologna, Italy; 4 Department of Optometry, University of Benin, Benin City 300238, Nigeria; 5 Africa Eye Laser Centre, Km 7, Benin City 300105, Nigeria; 6 Imperial College Ophthalmic Research Group (ICORG) Unit, Imperial College, 153-173 Marylebone Rd, London NW15QH, UK; 7 Department of Neurosciences, Reproductive Sciences and Dentistry, University of Naples Federico II, Via Pansini 5, 80131 Napoli, Italy; 8 Schepens Eye Research Institute of Mass Eye and Ear, Harvard Medical School, Boston, MA 02114, USA; 9 Department of Ophthalmology, Campus Bio-Medico University, 00128 Rome, Italy; 10 Department of Medicine and Surgery, University of Enna “Kore”, Piazza dell’Università, 94100 Enna, Italy; 11 Mediterranean Foundation “G.B. Morgagni”, 95125 Catania, Italy

**Keywords:** artificial intelligence, machine learning, deep learning, optic disc neuropathy, visual field, glaucoma

## Abstract

Background: If left untreated, glaucoma—the second most common cause of blindness worldwide—causes irreversible visual loss due to a gradual neurodegeneration of the retinal ganglion cells. Conventional techniques for identifying glaucoma, like optical coherence tomography (OCT) and visual field exams, are frequently laborious and dependent on subjective interpretation. Through the fast and accurate analysis of massive amounts of imaging data, artificial intelligence (AI), in particular machine learning (ML) and deep learning (DL), has emerged as a promising method to improve the early detection and management of glaucoma. Aims: The purpose of this study is to examine the current uses of AI in the early diagnosis, treatment, and detection of glaucoma while highlighting the advantages and drawbacks of different AI models and algorithms. In addition, it aims to determine how AI technologies might transform glaucoma treatment and suggest future lines of inquiry for this area of study. Methods: A thorough search of databases, including Web of Science, PubMed, and Scopus, was carried out to find pertinent papers released until August 2024. The inclusion criteria were limited to research published in English in peer-reviewed publications that used AI, ML, or DL to diagnose or treat glaucoma in human subjects. Articles were chosen and vetted according to their quality, contribution to the field, and relevancy. Results: Convolutional neural networks (CNNs) and other deep learning algorithms are among the AI models included in this paper that have been shown to have excellent sensitivity and specificity in identifying glaucomatous alterations in fundus photos, OCT scans, and visual field tests. By automating standard screening procedures, these models have demonstrated promise in distinguishing between glaucomatous and healthy eyes, forecasting the course of the disease, and possibly lessening the workload of physicians. Nonetheless, several significant obstacles remain, such as the requirement for various training datasets, outside validation, decision-making transparency, and handling moral and legal issues. Conclusions: Artificial intelligence (AI) holds great promise for improving the diagnosis and treatment of glaucoma by facilitating prompt and precise interpretation of imaging data and assisting in clinical decision making. To guarantee wider accessibility and better patient results, future research should create strong generalizable AI models validated in various populations, address ethical and legal matters, and incorporate AI into clinical practice.

## 1. Introduction

The world faces remarkable changes that affect society, welfare, health, and government policies. The global epidemic, social fear, and economic situation make the availability or supply of health services, especially for elective admissions, challenging and sometimes unattainable. This is why digital innovation should be beneficial in allowing the most significant number of people to access essential health services, even during the pandemic era of COVID-19. Ophthalmology must employ as many hi-tech devices as possible to reach the most patients needing eye care. New potential models of digital eye care, such as telemedicine, artificial intelligence (AI), and 5G technology, can be used to tackle and manage glaucoma and other forms of retinal neuropathies. Routinely, everyday ophthalmology deals with many images, data, and comparisons. First, the COVID-19 pandemic, now the war in Europe, and next, any other global social earthquake, will allow eye care to develop cutting-edge digital solutions and different ways to assist patients [1,2]. There is an urgent need to face and solve ethical issues about telemedicine and the application of AI in clinical eye care practice. 

Several fields of human life have been progressively involved in digital transformation. Therefore, ophthalmic services and the management of chronic eye diseases are managed by new health models supported by AI applications. Ocular neuropathies affect an increasing number of patients worldwide because of the aging of the population. Diagnostic imaging requests rapidly increase, but the number of ophthalmologists decreases globally. Thus, health professionals must use cutting-edge technology to meet patients’ expectations and perform their clinical tasks quickly. AI strategies facilitate feasibility, integration, and reporting of overwhelming optic disc imaging and testing. Intelligent automated systems will play a key role in screening, grading, and tailored therapy due to the unmanned assessment of disease activity, potential recurrences, timing of treatment, and possible retreatment.

A thorough search of pertinent databases, such as PubMed, Scopus, and Web of Science, was used to choose the papers for this study. The search was conducted from the reviews’ beginnings until August 2024. Included in the search phrases were “glaucoma”, “artificial intelligence”, “deep learning”, “machine learning”, “early detection”, “screening”, and “ophthalmology”. Boolean operators and filters were used to refine the search approach to find peer-reviewed publications, systematic reviews, and meta-analyses. The following inclusion criteria were used in the article selection process: research examining the application of artificial intelligence (AI), machine learning (ML), and deep learning (DL) to glaucoma early detection, screening, or diagnosis; English language publications; and papers published in journals with peer reviews. Among the exclusion standards were articles written in languages other than English; research not explicitly connected to artificial intelligence in ophthalmology or glaucoma; conference abstracts, editorials, opinion pieces, and publications that were not subjected to peer review; and research that lacked adequate information about the procedures or results of using AI to treat glaucoma. The titles and abstracts were checked for relevancy after the first search. After that, the inclusion and exclusion criteria were applied to the complete texts of any publications that might be relevant. Manuscripts were chosen for the final review based on the articles’ quality, contribution to the issue, and relevancy.

As we progress deeper into the era of digitalization, the incorporation of artificial intelligence (AI) into clinical disciplines such as ophthalmology is not merely an option but an essential requirement. Examining the use of AI in identifying glaucoma at an early stage lays the foundation for a more extensive conversation about how technology may transform the provision of healthcare and improve patient results. This study seeks to connect emerging AI technologies with their practical applications in ophthalmology, emphasizing the significance of innovation in tackling global healthcare concerns. This research has consequences that go beyond glaucoma. It provides insights that have the potential to completely transform the early diagnosis and treatment of various diseases.

The AI, DL, and ML models mentioned in this review primarily include the following:

- Convolutional Neural Networks (CNNs): extensively employed in numerous studies for the analysis of imaging data, including fundus pictures and optical coherence tomography (OCT) scans, exhibiting significant sensitivity and specificity in identifying glaucomatous alterations.
- Random Forests and Support Vector Machines (SVMs): these models are frequently employed for classification tasks, distinguishing between glaucomatous and healthy eyes based on certain clinical criteria, including retinal nerve fiber layer (RNFL) thickness and visual field (VF) test outcomes.
- Bayesian Networks: utilized in various instances to amalgamate several diagnostic tests and clinical data, yielding probabilistic results to evaluate glaucoma risk.
- Deep Learning Algorithms: models utilizing fundus imaging and OCT have demonstrated efficacy in the early detection and ongoing monitoring of glaucoma, particularly in recognizing anatomical alterations in the optic nerve head and retinal nerve fiber layer (RNFL).
- Explainable Artificial Intelligence (XAI): this is becoming an essential method for improving the interpretability of AI models, hence rendering clinical decision making more transparent and reliable for practitioners.

## 2. Methods

An extensive database search was conducted utilizing Web of Science, PubMed, and Scopus, using a combination of search terms including “glaucoma”, “artificial intelligence”, “machine learning”, “deep learning”, “early detection”, and “screening”. The search was performed until August 2024, including papers published from January 1990 to August 2024 and no language filters were implemented, except for limiting the results to English language articles.

Inclusion criteria included research that examined the application of artificial intelligence, machine learning, or deep learning for the diagnosis or treatment of glaucoma in human participants; articles published in peer-reviewed journals that provided adequate methodological detail for assessing the application of AI in clinical ophthalmology; and studies that offered original data or systematic reviews/meta-analyses pertinent to the utilization of AI in glaucoma diagnosis.

Exclusion criteria were based on studies not explicitly pertaining to AI, ML, or DL applications in glaucoma or ophthalmology; conference abstracts, opinion articles, and editorials lacking adequate data for analysis; and publications in languages other than English.

The subsequent quality criteria were used to evaluate the inclusion of publications. Methodological rigor was defined as research that exhibited a transparent approach to implementing AI, ML, or DL and was accompanied by sufficient documentation of model performance metrics (e.g., sensitivity, specificity, accuracy). Priority was assigned to research featuring substantial sample sizes and varied datasets that improved generalizability. We emphasized research that directly enhanced practical advancements in the diagnosis or management of glaucoma. All selected articles were published in peer-reviewed journals to guarantee academic integrity. These measures ensured that only the most pertinent and high-caliber publications were referenced in our paper, accurately representing the contemporary landscape of AI applications in glaucoma research.

## 3. Glaucoma

Glaucoma ranks as the second leading cause of blindness globally. It is characterized by the progressive neurodegeneration of retinal ganglion cells and permanent axonal loss from the optic nerve [3,4,5]. Globally, the prevalence of glaucoma is 3.4% for people aged 40–80, and it is anticipated that, by 2040, approximately 112 million people will be affected by glaucoma [6,7]. This optic nerve disease is characterized by the excavation and erosion of the neuro-retinal rim, clinically manifesting as increased optic nerve head cupping (ONH). Timely identification of optic nerve abnormalities and the subsequent evaluation of potential progressive structural and functional deterioration are essential for averting vision impairment and blindness [7].

OCT can detect initial small structural changes of ONH and macular ganglion cell density modifications. According to psychophysical data and age-matched normative comparisons, human professionals reckon that the thinning of ganglion fiber layers is an early sign of visual impairment caused by glaucoma [4]. Anyway, more analytical analysis of the large amount of data and parameters measured is currently lacking. AI application modalities might revolutionize the traditional screening and follow-up of the early stages of glaucoma. ML and DL can also identify new parameters to consider potential risk factors for blindness [8,9]. Nowadays, no DL algorithm is regarded as a trademark for automatically diagnosing and grading glaucoma’s severity, but research continues. 

Currently, the primary test to monitor visual function during glaucoma progression is the visual field (VF). However, a standard VF test is time-consuming and strongly dependent on the cooperation of a single patient. Modern DL algorithms are based on comparing VF tests, ONH images, and OCT scans, respectively, of peripapillary and macular ganglion cell axons [10]. These methods have shown exemplary performance in classifying glaucoma and detecting healthy eyes. The automated results allow ophthalmologists and glaucoma referees to quickly make better decisions during clinical practice [11,12].

Certain organizations have trained computer algorithms to identify glaucoma-like discs, characterized by vertical cup-to-disc ratios (CDR) of 0.7 and 0.8, respectively. Machine learning techniques have been utilized to distinguish glaucomatous nerve fiber layer damage from normal scans on wide-angle optical coherence tomography (9 × 12 mm) [11,12]. In the near future, DL applications could associate ONH defects with manifest visual field (VF) loss and automatically compare structural and functional optic nerve changes, respectively, learning from OCT and VF images, to detect the onset and the progression of glaucoma [13,14]. Other investigators have developed automated computer programs based on DL algorithms that recognize clinically relevant VF loss patterns, assigning a coefficient for each and comparing them over time [9,10,11]. This approach has been beneficial in detecting early partial and/or sectorial VF loss in patients and monitoring follow-up in people affected by glaucoma. Li and colleagues appraised the performance of a DL program to automatically recognize and classify glaucomatous ONH resulting from a comparative analysis of 48.116 color fundus photographs [13,14,15,16]. They observed that their DL system achieved referable glaucomatous optic neuropathy with an AUC of 0.986, a specificity of 92.0%, and a sensitivity of 95.6%. However, there were a lot of false negative results due to the presence of concurrent eye disorders or unique anatomical characteristics such as high myopia. Kim and colleagues tried to investigate ML models to predict glaucoma based on RNFL thickness and VF results [13,14,15]. In their study, the accuracy, specificity, sensitivity, and AUC were 0.98, 0.975, 0.983, and 0.979, respectively. Thus, this ML program was an exciting tool for discriminating between glaucoma and healthy eyes and a possible support for clinicians. Asaoka and colleagues created a DL model for the early diagnosis of glaucoma resulting from SD-OCT images, including both RNFL and ganglion cell complex thickness [15,16,17,18]. They proved that the AUC with their DL model was 93.7%, but, without pre-training, the AUC was significantly reduced between 76.6% and 78.8%.

The intricate nature of glaucoma as a medical condition, characterized by its gradual progression and ability to cause permanent vision impairment, emphasizes the crucial need for early detection. By comprehending the fundamental mechanics and identifying the components that contribute to risk, we may improve our understanding of AI’s significance to this particular domain. With the increasing global frequency of glaucoma, there is a growing need to create instruments that can accurately and promptly diagnose the condition. The subsequent sections will explore how AI is positioned to address this dilemma, providing renewed optimism for millions who are in danger.

## 4. Telemedicine, Deep Learning, Machine Learning, ChatGPT

Telemedicine provides remote ophthalmic services, which are essential during the COVID-19 pandemic, thereby minimizing unnecessary visits to distant and overcrowded hospitals. Telemedicine technologies, enabled by sophisticated communication networks and artificial intelligence (AI) analysis, have demonstrated significant potential for the early diagnosis of glaucoma in primary care environments, reducing unnecessary tests. [19]. The practical application of AI-based systems in real-world scenarios has proven the efficacy of evaluating fundus pictures in terms of sensitivity and specificity. Optical coherence tomography (OCT) is increasingly utilized for the follow-up of glaucoma, providing highly detailed images of the macula and optic disc. Artificial intelligence systems have been created to automatically detect illnesses using OCT pictures. Liu et al. used artificial intelligence techniques in a telemedicine platform to identify optic disc abnormalities [20]. In Shanghai, a densely populated city with a growing elderly population and a limited number of ophthalmologists, they introduced it to primary care facilities. 

The AI models demonstrate remarkable precision in referral selection and can identify conditions that threaten vision. The software significantly alleviated the workload of ophthalmologists by effectively recognizing and rejecting normal cases while simultaneously facilitating online consultations for pathological situations. Online consultations have been executed promptly, with most urgent cases being referred to secondary facilities [21]. 

Machine learning (ML), pioneered by Arthur Samuel in 1959, entails training software with extensive datasets to identify and comprehend particular patterns within the data. The primary concept involves the integration of multiple interconnected algorithms, each dedicated to finding specific characteristics. This system is termed a neural network as it seeks to emulate the functions of neurons in the human brain [22]. Deep learning (DL) is a subset of machine learning (ML) that employs multiple artificial neural networks (ANNs) organized in layers to emulate the processing functions of the human brain more precisely. Convolutional neural networks (CNNs) are a specialized type of artificial neural network (ANN) primarily employed to analyze pictures and videos. Successful data interpretation hinges on sensitivity, specificity, or the receiver operating characteristic curve. Over the past two decades, there has been a notable rise in the application of AI-driven methodologies, especially within the medical domain. Artificial intelligence and machine learning systems are often trained with digital images and quantitative data [23]. Deep learning has primarily been employed for medical imaging analysis in healthcare, specifically for ocular imaging techniques such as fundus retinographies, visual fields (VFs), and OCT scans. Deep learning may identify and track several ocular illnesses, including glaucoma, offering an alternative to traditional screening methods [24]. Despite numerous deep learning models being trained and tested on large datasets, additional research is necessary to explore external validation and generalization across varied demographics and imaging modalities. Deep learning has enabled the automated classification of excavated optic discs and the segmentation of retinal components in optical coherence tomography imaging with commendable precision [25]. This review provides details on the comparative performance of the AI models as follows:

Convolutional Neural Networks (CNNs)

- Fundus Photos: CNNs have exhibited robust efficacy in the analysis of fundus photos for the detection of glaucomatous optic neuropathy. Research indicates that CNN models can attain elevated accuracy, sensitivity, and specificity, frequently above 90%, particularly when substantial datasets are accessible for training.
- OCT Scans: Convolutional neural networks (CNNs) demonstrate efficacy when used with optical coherence tomography (OCT) data, offering intricate structural insights about the optic nerve head and retinal nerve fiber layer (RNFL). Convolutional neural networks (CNNs) have successfully identified early structural alterations in optical coherence tomography (OCT) scans, attaining AUC values between 0.93 and 0.98. Nonetheless, their performance is generally superior for images with distinctly characterized glaucomatous characteristics. The capacity of CNNs to identify nuanced differences in RNFL thickness enhances their efficacy in identifying glaucoma at an early stage by OCT.

Alternative Deep Learning (DL) Algorithms

- Visual Field (VF) Data: Machine learning models, encompassing deep learning, have been employed to forecast progression trends and evaluate functional vision impairment in glaucoma patients based on VF test data. These models can identify early visual field deterioration that may not be readily observable to doctors. Performance measures, namely AUC values ranging from **0.76 to 0.79**, have been documented for DL models identifying early VF progression. Although these values are inferior to those of structural imaging, they remain significant for clinical decision making.
- Multimodal Approaches: Certain research has utilized deep learning algorithms that amalgamate both structural (OCT) and functional (VF) data to enhance diagnostic precision. This multimodal strategy demonstrates potential by utilizing complementing information from several senses.

### Comparison of Data Types

Fundus Photographs against OCT Scans: CNNs excel in analyzing both fundus photos and OCT scans, with the main difference being the nature of the data presented. Fundus images are two-dimensional depictions, valuable for identifying significant optic nerve injury. Conversely, OCT delivers high-resolution three-dimensional pictures that elucidate the structural integrity of the retinal layers and optic nerve. Thus, CNNs evaluating OCT data may be more proficient in identifying early subtle glaucomatous alterations, especially in pre-perimetric glaucoma, where functional visual impairment has not yet been shown.

Limitations: Although CNNs are proficient in image-based diagnostics (fundus and OCT), their efficacy may be affected by the quality, diversity, and size of the dataset. Limited generalizability may arise from smaller datasets or those without heterogeneity, such as homogeneous populations. Moreover, whereas deep learning models exhibit great accuracy in identifying structural abnormalities, their capacity for elucidating predictions (i.e., decision-making transparency) continues to pose a difficulty.

CNNs and other deep learning algorithms excel in analyzing both fundus photographs and OCT scans; however, OCT data typically facilitate earlier identification of structural alterations, providing a superior advantage in the early detection of glaucoma. We have elucidated these aspects in the text to more effectively highlight the comparative advantages of each AI model for various glaucoma data types.

This review examines the function of AI models, specifically CNNs and DL algorithms, in analyzing OCT images to identify glaucomatous alterations. OCT offers cross-sectional imaging of the retina and optic nerve head, documenting essential characteristics such as retinal nerve fiber layer (RNFL) thickness and ganglion cell complex (GCC) integrity. These structural characteristics are crucial for the early identification of glaucoma.

AI algorithms, particularly CNNs, have been widely utilized for OCT data because they are proficient in identifying patterns inside intricate image collections. Research indicates that CNN-based models can evaluate OCT scans with exceptional precision, identifying even small structural damage linked to glaucoma. In some instances, these models surpass conventional diagnostic techniques for sensitivity and specificity. For example, CNNs utilized on OCT scans can attain very good AUC values, signifying robust diagnostic efficacy in detecting glaucomatous alterations.

Despite utilizing advanced technology for several aspects of eye care, including early disease identification and treatment monitoring, deep learning still needs further validation in clinical environments. Numerous barriers hinder the assimilation and prompt utilization of new technologies in medical practice. A major challenge is the reliability of training data collected from highly similar populations, leading to issues with the diversity of image characteristics and ethnic backgrounds. Enhancing the diversity of datasets may alleviate this challenge [26]. The lack of extensive datasets for rare diseases and ailments not frequently documented by imaging, such as cataracts, impedes model development. Moreover, the widespread use of AI models in healthcare is hindered by concerns regarding the opacity of these AI systems. Clinicians and patients simulate transparency in the classification of diseases by AI systems. Moreover, disparities in medico-legal considerations and regulatory authorizations among countries challenge implementation. The readiness of patients to embrace AI-based screening varies across different demographics and settings, thereby influencing the integration of this technology into clinical practice. Although numerous studies demonstrate a considerable degree of patient satisfaction, it is crucial to acknowledge that cultural factors may affect acceptability and pose hurdles to applying these findings. It is imperative to resolve these difficulties to fully actualize the potential of AI in ophthalmology and seamlessly integrate it into clinical practice for improved patient care [27]. ChatGPT, an AI language model developed by OpenAI, possesses the capacity to significantly impact public health. ChatGPT uses extensive datasets to generate text that closely mimics human speaking. This may aid individuals and communities in making educated decisions regarding their health. ChatGPT possesses various potential applications in the healthcare sector, such as disseminating information on public health issues, responding to inquiries regarding health promotion and disease prevention strategies, recognizing the contributions of community health workers, examining the impact of social and environmental determinants on community health, and offering information about community health programs and services. Nonetheless, ChatGPT has numerous public health limitations, including restricted accuracy, data limitations and biases, minimal user engagement, lack of contextual comprehension, and absence of direct interaction with healthcare professionals. Consequently, it is imperative to acknowledge these limitations, and that ChatGPT and other tools are utilized to ensure accurate public health outcomes [28]. The amalgamation of AI and telemedicine in ophthalmology represents a significant evolution in our methodology for patient care. This initiative not only guarantees the accessibility of eye care but also improves its efficiency and customization. As we increasingly integrate these technologies, it is essential to consider their potential for optimization to serve diverse patient populations, especially those in rural or underserved areas. The application of AI in telemedicine can potentially transform healthcare delivery profoundly, and if implemented successfully, it might serve as a model for other medical fields.

## 5. Advantages of Artificial Intelligence in Glaucoma Management

There are several potential advantages of employing AI in glaucoma management and screening. With regards to accuracy, AI methods (CNNs, DL, ML), especially CNNs and DL algorithms, have exhibited significant accuracy in identifying glaucomatous alterations from imaging techniques such as fundus photographs and OCT scans. Numerous research studies indicate that AI models have attained AUCs, sensitivities, and specificities that are beyond 90%. These techniques are proficient in identifying glaucoma at an early stage by examining minor structural alterations in the optic nerve and retinal nerve fiber layer (RNFL), which traditional methods may overlook. Standard diagnostic techniques, including visual field (VF) testing, intraocular pressure (IOP) measurement, and manual analysis of **OCT and fundus photography, depend significantly on the subjective interpretation of physicians. Although these methods are well established, their accuracy may fluctuate based on the examiner’s expertise and the patient’s compliance, especially in visual field examinations. Conventional techniques typically identify glaucoma at a later stage of the disease when substantial damage has already transpired.

Concerning efficiency, AI models exhibit exceptional efficiency in processing extensive imaging data, facilitating expedited diagnoses without the necessity for laborious manual evaluations. Artificial intelligence can automate standard screening procedures, substantially alleviating the burden on physicians and facilitating the earlier identification of glaucoma. AI-driven systems may evaluate OCT scans or fundus photographs in a few seconds while concurrently delivering reliable and reproducible outcomes. This enables enhanced scalability, especially in telemedicine and extensive screening initiatives. Conventional methods and traditional glaucoma detection techniques, including manual imaging analysis and visual field assessments, are more time-intensive. Visual field testing is contingent upon patient compliance and may require 15 to 30 min per eye, resulting in inefficiencies in clinical environments. Furthermore, traditional procedures frequently necessitate retesting for validation, hence prolonging the duration needed for diagnosis and care.

When considering cost effectiveness, after training and implementation, AI systems can provide enduring cost reductions, especially in high-volume screening contexts. Through the automation of the detection process, AI diminishes the necessity for recurrent visits and protracted diagnostic procedures, hence enhancing overall resource efficiency. Furthermore, AI’s capacity to identify glaucoma in its first stages can facilitate prompt therapies, thereby decreasing the long-term expenses associated with managing advanced conditions. The initial expenditures for AI systems, encompassing software, infrastructure, and training, may be substantial but are mitigated by diminished labor costs and enhanced efficiency over time. Conventional glaucoma detection procedures, although initially less expensive, may incur significant costs over time due to the necessity for frequent follow-up appointments, repeated examinations, and extended periods for physicians to analyze results. Moreover, traditional approaches may yield delayed diagnoses, potentially resulting in increased long-term treatment expenses as the disease advances to more severe stages. In resource-constrained environments, the availability of a sophisticated diagnostic apparatus may serve as a constraint, rendering AI a more scalable and economically viable option over time.

AI methodologies provide substantial benefits over traditional glaucoma detection methods in terms of precision, efficiency, and economic viability. AI’s capacity to automate extensive screening and accurately identify glaucoma at an early stage renders it an invaluable asset in clinical practice and population-level glaucoma management. Although the initial installation expenses of AI systems may be substantial, their long-term advantages in enhanced diagnostic precision and resource optimization render them preferable to conventional methods.

To sum up, there are several potential advantages of employing AI in glaucoma management and screening. With regards to accuracy, AI methods (CNNs, DL, ML), especially CNNs and DL algorithms, have exhibited significant accuracy in identifying glaucomatous alterations from imaging techniques such as fundus photographs and OCT scans. Numerous research studies indicate that AI models have attained AUCs, sensitivities, and specificities that are beyond 90%. These techniques are proficient in identifying glaucoma at an early stage by examining minor structural alterations in the optic nerve and retinal nerve fiber layer (RNFL), which traditional methods may overlook. Standard diagnostic techniques, including visual field (VF) testing, intraocular pressure (IOP) measurement, and manual analysis of **OCT and fundus photography, depend significantly on the subjective interpretation of physicians. Although these methods are well established, their accuracy may fluctuate based on the examiner’s expertise and the patient’s compliance, especially in visual field examinations. Conventional techniques typically identify glaucoma at a later stage of the disease when substantial damage has already transpired.

## 6. Discussion

Cutting-edge technology facilitates the acquisition of a significant amount of imaging data. Consequently, AI is expected to progress among the majority of healthcare practitioners across several medical disciplines. The accurate acquisition and analysis of images are essential for achieving a precise diagnosis and identifying the most appropriate treatment plan. Progress in computational resources and machine learning algorithms may improve the clinical practice of physicians and human specialists. This may result in an exceptionally accurate, reliable, and consistent diagnostic technique exhibiting high specificity and sensitivity [29]. Other than accuracy, efficiency (particularly the time required for each test) is a critical demand for most glaucoma specialists. Conversely, the increasing population of senior adults in the community will result in a global rise in retinal diseases. Simultaneously, the accessibility of ophthalmologists and specialty eye clinics would be constrained. The expected increase in the availability of medical imaging resources and the reduction in computer technology costs make AI aid essential. Moreover, the extensive variability in the interpretation of imaging results by various examiners and the resulting discrepancies in consensus among retinal specialists is another factor to consider [30]. We are assured that the opportunity to integrate artificial intelligence into routine clinical practice is promising. Deep learning is anticipated to impact clinical practice significantly. Recent ophthalmological research has demonstrated the validity and accuracy of deep learning algorithms for the early identification, continuous monitoring, and focused therapy of retinal illnesses, including glaucoma [31]. In recent years, deep learning has demonstrated efficacy in various applications, including statistical analysis, signal processing, pattern recognition, and image processing. Consequently, it is expected to provide enhanced innovative support for research and applications in ophthalmology. In the domain of medical retina, extensive datasets are now accessible for artificial intelligence models, particularly fundus images and OCT scans. These datasets are essential for training deep learning algorithms and creating automated detection systems for the early development of illnesses such as glaucoma. The precision of these devices is analogous to that of retinal specialists [31,32]. There is a prevailing agreement that these technologies will be utilized for prescreening or to augment the efforts of human specialists through decision support systems. Computer algorithms are expected to provide a more objective and reproducible assessment of retinal morphology and associated alterations, hence aiding in the analysis of disease progression. Ophthalmologists will receive accurate assistance in deciding whether to initially treat or re-treat macular edema with intravitreal injections based on its progression and alterations observed through imaging analyzed by automated deep learning algorithms. Due to extensive and sustained collaborations between AI laboratories and eye centers, AI is now acquiring the capability to analyze and potentially integrate the vast amounts of data derived from fundus images, visual field tests (VFs), and optical coherence tomography (OCT) scans into clinical trials. Knowledge is derived from the learning process using digital data gathered from extensive datasets, and experiments validate the precision of AI-assisted diagnosis. Nevertheless, using intricate mathematical algorithms enables AI models to identify glaucoma, customize appropriate medical interventions, and strategize the most advantageous intervals for subsequent treatments based on individual biomarkers. In addition, illness models based on artificial intelligence are anticipated to provide a valuable understanding of the underlying mechanisms of glaucoma by identifying and analyzing microstructural biomarkers related to the retina or optic disc morphology. This might potentially result in the creation of virtual models and data simulations [33].

Although this work provides a comprehensive overview of artificial intelligence’s current and future applications in glaucoma diagnosis, it is essential to acknowledge a few limitations. The primary limitation is the heterogeneity of the included research, which varies in terms of the clinical outcomes investigated, study methodology, sample size, and artificial intelligence models utilized. Due to the heterogeneity, it is challenging to make direct comparisons of study results or reach a definitive conclusion on the superiority of AI strategies. Additionally, concerns arise regarding the applicability of findings to more varied patient populations because of the limited evaluation of AI models on predominantly homogeneous individuals.

Another limitation is the utilization of retroactive data, which may be subject to biases inherent in the data sources. Moreover, many AI models are considered “black boxes”, indicating that their decision-making processes are not entirely transparent, posing challenges for physicians in accepting and relying on them. This article highlights the significance of conducting further large-scale prospective clinical studies to evaluate AI algorithms in diverse therapeutic settings prior to their widespread use. Furthermore, there remain unresolved inquiries and a requirement for further investigation regarding the ethical, legal, and data privacy issues associated with the implementation of AI in healthcare, particularly in the field of ophthalmology.

The future outlook for utilizing AI in identifying and treating glaucoma is quite promising. To enhance the applicability of AI models across different populations, future research should focus on developing and validating them using large and varied datasets. Prospective research to ascertain the therapeutic value and effectiveness of AI algorithms in real-world scenarios is necessary to improve confidence among clinicians and patients. Furthermore, it is imperative to clarify the enigmatic nature of these models.

AI can also be employed in routine screening programs, particularly in disadvantaged regions with limited availability of ophthalmology experts. The advancement of artificial intelligence (AI) alongside enhancements in telemedicine and imaging technologies has the potential to enable the early and more precise detection of glaucoma, hence reducing the worldwide occurrence of the condition. Furthermore, to address the ethical, legal, and societal consequences and ensure the security, effectiveness, and impartiality of AI technologies for all patients, it is crucial to have multidisciplinary collaboration among AI developers, physicians, and regulators. Some AI technologies are summarized in Table 1 below.

The application of AI models in glaucoma detection showcases the remarkable progress made in this field. While still evolving, these models have already demonstrated their potential to enhance diagnostic accuracy and efficiency. Moving forward, the focus should be refining these models to ensure they are robust, reliable, and applicable across various clinical settings. As AI advances, it will be crucial to maintain a balance between innovation and practicality, ensuring that these technologies complement rather than complicate the clinical workflow.

Like other innovative technologies, the incorporation of AI into clinical practice presents its own unique difficulties. Nevertheless, these obstacles should be perceived as chances for development and enhancement. By tackling concerns such as the transparency of algorithms, including diverse data and ethical considerations, we may create a path for artificial intelligence to become an essential and irreplaceable instrument in the healthcare field. The promise of AI in ophthalmology is exciting, but its realization necessitates meticulous planning, interdisciplinary collaboration, and a dedication to ongoing enhancement.

Although AI holds great potential in improving the identification and treatment of glaucoma, several limitations and drawbacks must be resolved to thoroughly harness its capabilities. A significant constraint is the exorbitant expense linked to the implementation and upkeep of AI systems. These technologies necessitate sophisticated up-to-date technology and software, which can be excessively costly for several healthcare practitioners, especially in low-resource environments. Furthermore, incorporating artificial intelligence (AI) into clinical practice necessitates a substantial commitment to training and education for healthcare professionals. They must acquire the necessary skills to utilize these novel tools proficiently. The high level of difficulty in learning can hinder mainstream acceptance, especially among senior professionals who may have little knowledge of digital technologies [41]. 

Another notable obstacle is the reliance on state-of-the-art technology, which requires regular updates to maintain effectiveness. As artificial intelligence algorithms progress, the gear and software that assist them must also advance, resulting in ongoing expenses and interruptions in clinical procedures. Dependence on technology also gives rise to worries over the digital gap, wherein only well-endowed institutions may reap the advantages of the most recent innovations, thereby exacerbating discrepancies in healthcare accessibility and quality. Moreover, deploying AI systems necessitates a resilient data infrastructure, which may not be accessible in every clinical setting, particularly in rural or underprivileged regions. 

One particularly problematic aspect of the growing dependence on AI in diagnosis is the potential degradation of traditional clinical skills. With the increasing proficiency of AI systems in analyzing complicated data and making diagnostic judgments, there is a concern that certain aspects of medical practice, such as semiotics and purely clinical talents, which were traditionally considered essential, may be given less importance in medical education. For example, the intricate skill of conducting thorough patient examinations and the capacity to make diagnoses solely based on clinical indicators may receive less emphasis. This transition may result in a cohort of doctors who possess advanced technological abilities but may lack proficiency in providing direct patient care, potentially compromising the quality of the doctor–patient interaction.

Furthermore, the growing capacity of artificial intelligence (AI) to identify medical diseases without requiring direct engagement with patients gives rise to ethical and professional apprehensions. There is a risk that upcoming medical professionals could excessively depend on AI tools, compromising their clinical judgment and decision-making abilities. This may lead to a healthcare system in which the human aspect of medicine is not important enough, with physicians primarily serving as mediators between patients and machines rather than as comprehensive caregivers. It is essential to maintain the emphasis on clinical acumen in medical school, even in an environment boosted by artificial intelligence, to preserve the fundamental nature of medical practice.

To summarize, although AI can revolutionize ophthalmology and other medical domains, these progressions are accompanied by substantial obstacles. To ensure that AI improves healthcare quality, addressing the issues of high costs, reliance on ever-changing technology, and the possibility of future physicians losing their skills is crucial. Finding a middle ground between adopting technology advancements and maintaining the fundamental skills that characterize effective medical care as we progress is vital. The future of AI in healthcare hinges not just on technological progress but also on our capacity to conscientiously and prudently incorporate these tools into clinical training and practice.

## 7. Conclusions

To recapitulate, prompt identification and quick intervention for neuropathy are essential to avert significant vision impairment and enhance one’s quality of life. The integration of AI systems has the potential to significantly affect routine diagnosis methods and change treatment and retreatment intervals for retinal or neurological illnesses. This is especially true when considering the availability of advanced automated retinal imaging technologies.

Further investigation into artificial intelligence (AI) is necessary to thoroughly evaluate ethical difficulties, provide data protection, mitigate biases in patient demographics, defend intellectual property, improve cybersecurity, establish accountability, and resolve regulatory issues. Impartiality is an essential element. Alongside the necessity for technological precision and replicability, it is imperative to ensure that AI is utilized to mitigate societal inequities and barriers, facilitating unobstructed access to healthcare facilities and programs. Given the recent increase in glaucoma cases, it is essential to integrate AI technologies into existing healthcare frameworks. These technologies seek to enhance the early identification of disease onset and establish a robust basis for individualized medical therapy. In conclusion, the intersection of AI and ophthalmology represents a significant leap forward in our ability to detect and manage glaucoma. While there are still hurdles to overcome, the progress made thus far is encouraging. As we look to the future, we must continue exploring new AI applications, refining existing models, and ensuring that these technologies are accessible to all. The journey toward fully integrating AI into clinical practice is just beginning, but with continued effort and innovation, it holds the promise of dramatically improving patient outcomes worldwide.

## Figures and Tables

**Table 1:** Novel AI technology is used to diagnose glaucoma.

AI Method	Description	Application	Key Features
Deep Learning (DL) on Fundus Images [34]	It uses convolutional neural networks (CNNs) to analyze retinal images.	Early detection and classification of glaucoma.	High accuracy and non-invasive, can be used for mass screening.
Optical Coherence Tomography (OCT) Imaging [35,36]	AI algorithms analyze OCT scans to detect structural changes in the optic nerve head and retinal nerve fiber layer.	Diagnosis and monitoring of glaucoma progression.	High sensitivity and specificity, detailed structural analysis.
Visual Field (VF) Testing [37]	Machine learning models predict visual field loss patterns.	Functional assessment of glaucoma.	Can detect progression earlier than conventional methods.
Stereo Fundus Imaging [38]	Combines 2D images from different viewpoints to create a 3D view of the fundus.	Screening and diagnosis of glaucoma.	Provides a comprehensive view of the optic nerve head.
Bayesian Networks [39]	Uses probabilistic models to integrate various diagnostic tests and clinical data.	Comprehensive risk assessment and diagnosis.	Integrates multiple data sources and provides probabilistic outcomes.
Explainable AI (XAI) [40]	AI models that provide transparent and interpretable results.	Enhancing clinician trust and decision making.	Improves understanding of AI decisions and regulatory compliance.

## References

[1] Baxter S.L., Marks C., Kuo T.T., Ohno-Machado L., Weinreb R.N. (2019). Machine Learning-Based Predictive Modeling of Surgical Intervention in Glaucoma Using Systemic Data from Electronic Health Records. Am. J. Ophthalmol..

[2] Baxter S.L., Saseendrakumar B.R., Paul P., Kim J., Bonomi L., Kuo T.T., Loperena R., Ratsimbazafy F., Boerwinkle E., Cicek M. (2021). Predictive Analytics for Glaucoma Using Data from the All of Us Research Program. Am. J. Ophthalmol..

[3] Jonas J.B., Aung T., Bourne R.R., Bron A.M., Ritch R., Panda-Jonas S. (2017). Glaucoma. Lancet.

[4] Allison K., Patel D., Alabi O. (2020). Epidemiology of Glaucoma: The Past, Present, and Predictions for the Future. Cureus.

[5] Quigley H.A., Broman A.T. (2006). The number of people with glaucoma worldwide in 2010 and 2020. Br. J. Ophthalmol..

[6] Tham Y.C., Li X., Wong T.Y., Quigley H.A., Aung T., Cheng C.Y. (2014). Global prevalence of glaucoma and projections of glaucoma burden through 2040: A systematic review and meta-analysis. Ophthalmology.

[7] Rosenberg L.F. (1995). Glaucoma: Early detection and therapy for prevention of vision loss. Am. Fam. Physician.

[8] Lucy K.A., Wollstein G. (2016). Structural and Functional Evaluations for the Early Detection of Glaucoma. Expert Rev. Ophthalmol..

[9] Gandhi M., Dubey S. (2013). Evaluation of the Optic Nerve Head in Glaucoma. J. Curr. Glaucoma Pract..

[10] Christopher M., Belghith A., Weinreb R.N., Bowd C., Goldbaum M.H., Saunders L.J., Medeiros F.A., Zangwill L.M. (2018). Retinal Nerve Fiber Layer Features Identified by Unsupervised Machine Learning on Optical Coherence Tomography Scans Predict Glaucoma Progression. Investig. Ophthalmol. Vis. Sci..

[11] Elze T., Pasquale L.R., Shen L.Q. (2015). Patterns of functional vision loss in glaucoma determined with archetypal analysis. J. R. Soc. Interface.

[12] Wang M., Pasquale L.R., Shen L.Q., Boland M.V., Wellik S.R., De Moraes C.G., Myers J.S., Wang H., Baniasadi N., Li D. (2018). Reversal of Glaucoma Hemifield Test Results and Visual Field Features in Glaucoma. Ophthalmology.

[13] Tanna A.P., Bandi J.R., Budenz D.L., Feuer W.J., Feldman R.M., Herndon L.W., Rhee D.J., Whiteside-de Vos J. (2011). Interobserver agreement and intraobserver reproducibility of the subjective determination of glaucomatous visual field progression. Ophthalmology.

[14] Viswanathan A.C., Crabb D.P., McNaught A.I., Westcott M.C., Kamal D., Garway-Heath D.F., Fitzke F.W., Hitchings R.A. (2003). Interobserver agreement on visual field progression in glaucoma: A comparison of methods. Br. J. Ophthalmol..

[15] Li Z., He Y., Keel S., Meng W., Chang R.T., He M. (2018). Efficacy of a Deep Learning System for Detecting Glaucomatous Optic Neuropathy Based on Color Fundus Photographs. Ophthalmology.

[16] Ting D.S.W., Cheung C.Y., Lim G., Tan G.S.W., Quang N.D., Gan A., Hamzah H., Garcia-Franco R., San Yeo I.Y., Lee S.Y. (2017). Development and Validation of a Deep Learning System for Diabetic Retinopathy and Related Eye Diseases Using Retinal Images from Multiethnic Populations with Diabetes. JAMA.

[17] Yousefi S., Kiwaki T., Zheng Y., Sugiura H., Asaoka R., Murata H., Lemij H., Yamanishi K. (2018). Detection of Longitudinal Visual Field Progression in Glaucoma Using Machine Learning. Am. J. Ophthalmol..

[18] Kass M.A., Heuer D.K., Higginbotham E.J., Johnson C.A., Keltner J.L., Miller J.P., Parrish R.K., Wilson M.R., Gordon M.O. (2002). The Ocular Hypertension Treatment Study: A randomized trial determines that topical ocular hypotensive medication delays or prevents the onset of primary open-angle glaucoma. Arch. Ophthalmol..

[19] Nguyen H.V., Tan G.S., Tapp R.J., Mital S., Ting D.S., Wong H.T., Tan C.S., Laude A., Tai E.S., Tan N.C. (2016). Cost-effectiveness of a National Telemedicine Diabetic Retinopathy Screening Program in Singapore. Ophthalmology.

[20] Liu X., Zhao C., Wang L., Wang G., Lv B., Lv C., Xie G., Wang F. (2022). Evaluation of an OCT-AI-Based Telemedicine Platform for Retinal Disease Screening and Referral in a Primary Care Setting. Transl. Vis. Sci. Technol..

[21] Jill Hopkins J., Keane P.A., Balaskas K. (2020). Delivering personalized medicine in retinal care: From artificial intelligence algorithms to clinical application. Curr. Opin. Ophthalmol..

[22] LeCun Y., Bengio Y., Hinton G. (2015). Deep learning. Nature.

[23] Dixit A., Yohannan J., Boland M.V. (2021). Assessing Glaucoma Progression Using Machine Learning Trained on Longitudinal Visual Field and Clinical Data. Ophthalmology.

[24] Ting D.S.W., Lin H., Ruamviboonsuk P., Wong T.Y., Sim D.A. (2020). Artificial intelligence, the internet of things, and virtual clinics: Ophthalmology at the digital translation forefront. Lancet Digit. Health.

[25] Shi N.N., Li J., Liu G.H., Cao M.F. (2024). Artificial intelligence for the detection of glaucoma with SD-OCT images: A systematic review and Meta-analysis. Int. J. Ophthalmol..

[26] Mayro E.L., Wang M., Elze T., Pasquale L.R. (2020). The impact of artificial intelligence in the diagnosis and management of glaucoma. Eye.

[27] Biswas S.S. (2023). Role of Chat GPT in Public Health. Ann. Biomed. Eng..

[28] Nazir F., Jawed S., Tariq S.M. (2023). Chat GPT and its potential role in medicine. J. Pak. Med. Assoc..

[29] Singh L.K., Pooja, Garg H., Khanna M. (2022). Performance evaluation of various deep learning based models for effective glaucoma evaluation using optical coherence tomography images. Multimed. Tools Appl..

[30] An G., Omodaka K., Hashimoto K., Tsuda S., Shiga Y., Takada N., Kikawa T., Yokota H., Akiba M., Nakazawa T. (2019). Glaucoma Diagnosis with Machine Learning Based on Optical Coherence Tomography and Color Fundus Images. J. Healthc. Eng..

[31] Lisboa R., Paranhos A., Weinreb R.N., Zangwill L.M., Leite M.T., Medeiros F.A. (2013). Comparison of different spectral domain OCT scanning protocols for diagnosing preperimetric glaucoma. Investig. Ophthalmol. Vis. Sci..

[32] Asaoka R., Murata H., Iwase A., Araie M. (2016). Detecting Preperimetric Glaucoma with Standard Automated Perimetry Using a Deep Learning Classifier. Ophthalmology.

[33] Huang X., Islam M.R., Akter S., Ahmed F., Kazami E., Serhan H.A., Abd-Alrazaq A., Yousefi S. (2023). Artificial intelligence in glaucoma: Opportunities, challenges, and future directions. Biomed. Eng. Online.

[34] Coan L.J., Williams B.M., Krishna Adithya V., Upadhyaya S., Alkafri A., Czanner S., Venkatesh R., Willoughby C.E., Kavitha S., Czanner G. (2023). Automatic detection of glaucoma via fundus imaging and artificial intelligence: A review. Surv. Ophthalmol..

[35] Zapata M.A., Royo-Fibla D., Font O., Vela J.I., Marcantonio I., Moya-Sánchez E.U., Sánchez-Pérez A., Garcia-Gasulla D., Cortés U., Ayguadé E. (2020). Artificial Intelligence to Identify Retinal Fundus Images, Quality Validation, Laterality Evaluation, Macular Degeneration, and Suspected Glaucoma. Clin. Ophthalmol..

[36] Zeppieri M., Marsili S., Enaholo E.S., Shuaibu A.O., Uwagboe N., Salati C., Spadea L., Musa M. (2023). Optical Coherence Tomography (OCT): A Brief Look at the Uses and Technological Evolution of Ophthalmology. Medicina.

[37] Wang M., Shen L.Q., Pasquale L.R., Boland M.V., Wellik S.R., De Moraes C.G., Myers J.S., Nguyen T.D., Ritch R., Ramulu P. (2020). Artificial Intelligence Classification of Central Visual Field Patterns in Glaucoma. Ophthalmology.

[38] Liu Y., Yip L.W.L., Zheng Y., Wang L. (2022). Glaucoma screening using an attention-guided stereo ensemble network. Methods.

[39] Tucker A., Vinciotti V., Liu X., Garway-Heath D. (2005). A spatio-temporal Bayesian network classifier for understanding visual field deterioration. ArtifIntell. Med..

[40] Oh S., Park Y., Cho K.J., Kim S.J. (2021). Explainable Machine Learning Model for Glaucoma Diagnosis and Its Interpretation. Diagnostics.

[41] Wang C.-Y., Nguyen H.-T., Fan W.-S., Lue J.-H., Saenprasarn P., Chen M.-M., Huang S.-Y., Lin F.-C., Wang H.-C. (2024). Glaucoma Detection through a Novel Hyperspectral Imaging Band Selection and Vision Transformer Integration. Diagnostics.

